# Spinal Versus General Anesthesia for Lumbar Discectomy: Patient-Centered Analysis of Satisfaction with Anesthesia Service †

**DOI:** 10.3390/medicina62030524

**Published:** 2026-03-12

**Authors:** Marius Rimaitis, Diana Bilskienė, Kęstutis Rimaitis, Indrė Cirkelė, Andrius Macas

**Affiliations:** 1 Department of Anesthesiology, Lithuanian University of Health Sciences, LT-44307 Kaunas, Lithuania; diana.bilskiene@lsmu.lt (D.B.); kestutis.rimaitis@lsmu.lt (K.R.); andrius.macas@lsmu.lt (A.M.); 2 Department of Nursing, Lithuanian University of Health Sciences, LT-44307 Kaunas, Lithuania; indrecirkele@gmail.com

**Keywords:** lumbar discectomy, spinal versus general anesthesia, satisfaction

## Abstract

*Background and Objectives:* Spinal (SA) and general anesthesia (GA) are both available for lumbar disc hernia (LDH) surgery. Patient satisfaction with anesthesia service is under-investigated and may help identify areas requiring improvement, leading to better care. *Materials and Methods:* A prospective, non-randomized, survey-based study was performed in patients who underwent LDH surgeries under SA or GA. Patients rated perioperative pain (preoperative and postoperative days (PODs) 0, 1, and 2) and satisfaction with perioperative care (10 questions) on a numeric rating scale (NRS) from 0 to 10, and an overall satisfaction score (OSS) was calculated; a patient discomfort questionnaire was also used. Study outcomes were pain scores, satisfaction with care, and discomfort reported by SA and GA patients. *Results:* In total, 209 completed questionnaires in the GA and SA groups (114 vs. 95) were available for final analysis. Baseline characteristics did not differ significantly between the two groups. The proportion of patients with severe pain decreased from >80% preoperatively to 6% on POD2, and pain scores did not differ significantly between groups. Mean overall satisfaction scores (OSSs) were high: 9.71 (maximum OSS: 57% of cases) in the GA group, and 9.75 (maximum OSS: 53.7% of cases) in the SA group (*p* = 0.95). The ceiling effect of the patient satisfaction questionnaire had to be addressed. There was no association between the type of anesthesia and OSS. Sources of discomfort were similar between groups, except for oropharyngeal discomfort being more prevalent in the GA group (*p* < 0.05). Postoperative pain was reported as a source of discomfort by >50% of patients in both the SA and GA groups. Regression analysis identified anxiety and nude body exposure as preoperative factors associated with decreased satisfaction with anesthesia. Postoperative factors associated with submaximal satisfaction were PONV, cold, mouth dryness, and pain. Pain on POD 0 did not influence overall patient satisfaction. An association was only found when pain persisted on POD 1 and POD 2. *Conclusions:* No significant differences between the two anesthesia methods were found. Patient information, anxiety management, and privacy protection are important for patient satisfaction. In the postoperative period, pain and PONV management must be equally addressed, irrespective of the anesthesia method used. Further efforts to develop optimal tools for patient satisfaction assessment are necessary.

## 1. Introduction

Intervertebral disc hernia is a common spinal pathology associated with adverse impacts on the patient’s quality of life and is a significant burden to health care systems. Initial treatment options are conservative, but a substantial fraction of patients eventually undergo surgery. While complex spinal surgeries require general anesthesia (GA), intervertebral disc hernias predominantly occur in the lower back and can be successfully managed under spinal anesthesia (SA). Both anesthesia methods are reported to be effective and ensure satisfactory surgical conditions and patient comfort [1,2,3,4]. SA has been associated with lower risk of patient positioning-related injuries and postoperative pulmonary complications, as well as better postoperative pain management and lower costs [5]. Previous meta-analyses have demonstrated significantly lower immediate postoperative pain scores [4]. GA, on the other hand, has the advantage of unlimited duration, airway control, complete immobility of the patient, and the possibility of checking for neurological deficits immediately after the surgery [1,2,3,4,6,7,8,9].

Patient satisfaction is an important marker of the overall quality of health care service provided [10]. However, studies investigating the impact of the chosen anesthesia method on perioperative pain control, overall comfort, and satisfaction with anesthesia service following intervertebral disc hernia surgery from the patient perspective are still lacking.

Patient-centered analysis of satisfaction with anesthesia service may provide more insights into selecting the optimal anesthesia approach for lumbar disc hernia surgery, help in identifying areas requiring improvement, and lead to better care.

## 2. Materials and Methods

### 2.1. Patient Population

A single-center, prospective, observational, structured survey-based study was performed at a tertiary neurosurgical center.

Patient inclusion criteria: Adult patients, elective lumbar microdiscectomies (L3/4, L4/5, or L5/S1), both spinal (SA) and general anesthesia (GA) considered acceptable management options. Exclusion criteria: Patient refusal to participate, surgery including multiple intervertebral spaces or any interspace above L3/4, spinal fusion. All eligible patients were informed about the study upon admission. The ultimate choice of anesthesia method was based on consensus between the patient and the anesthesiologist, who was unaware of the study.

Population-based calculations identified that we needed to include at least 220 patients to make reasonable calculations [11]. Following written consent, from 10 January to 31 July 2023, a total of 230 patients participated in the study. The anonymous questionnaires were presented upon patient admission and filled out by the subjects themselves.

Primary study outcome: Patient-reported satisfaction with perioperative care in SA and GA groups. Secondary outcomes: Postoperative pain scores, satisfaction with different aspects of perioperative care, and discomfort experienced by patients in SA and GA groups.

### 2.2. Anesthesia

Spinal anesthesia (SA) was performed in the lateral decubitus position under light sedation with intravenous (IV) midazolam 1 to 5 mg, at the L3/4 or L4/5 interspace with a 27G pencil point spinal needle (B. Braun, Melsungen, Germany). Following identification of the subarachnoid space by the free flow of cerebrospinal fluid, 2.8 to 3.4 mL of isobaric 0.5% spinal bupivacaine was injected. The patient was subsequently positioned in the prone position, and intravenous propofol infusion was titrated to maintain moderate sedation (25–50 mcg/kg/min) throughout the surgery. No additional medications were routinely used in the SA group. General anesthesia (GA) was induced with propofol 2 to 2.5 mg/kg, fentanyl 1 mcg/kg, and rocuronium 0.6 mg/kg and maintained with sevoflurane 1.0 minimum alveolar concentration. Following tracheal intubation, confirmation of tube placement, and protection of the compression sites, the patient remained prone for the length of surgery. GA patients were routinely given dexamethasone 4 mg and ondansetron 8 mg for nausea and vomiting prophylaxis, as well as ketoprofen 1–2 mg/kg and paracetamol 1 g IV for postoperative analgesia. Following surgery, all patients were awakened in the operating room and transferred to the post-anesthesia care unit with stable vital signs.

### 2.3. Research Instruments

The questionnaire consisted of two parts. The first part included basic demographic data, medical history, and questions about the discomfort experienced and pain management from the perioperative period until postoperative day 2 (see Supplementary Material).

The second part assessed patient satisfaction with the service, which was quantified based on the patient satisfaction questionnaire presented by Capuzzo et al. (Table 1), used with permission from the author [12,13]. Each patient had to rate their satisfaction with different aspects of perioperative care on a numeric rating scale (NRS) from 0 (no satisfaction) to 10 (the maximum satisfaction possible); their overall satisfaction score (OSS) was calculated from a sum of ratings to all questions divided by the number of questions (OSS = SUM (q1:q10)/10). Based on numeric rating scale evaluations, pain was classified as mild (NRS 0–2), moderate (NRS 3–6), or severe (NRS 7–10). High satisfaction with care was considered as NRS 8–10, whereas patients reporting NRS < 8 were considered as not fully satisfied [14].

### 2.4. Data Analysis

The data were analyzed with SPSS Statistics version 25.0 (IBM SPSS, Armonk, NY, USA). The normality of quantitative data was assessed using the Kolmogorov–Smirnov test. Normally distributed quantitative variables of two independent samples were compared using Student’s *t*-test. Quantitative variables of not normal distribution were compared using the Mann–Whitney *U*-test and Wilcoxon signed-rank test. The chi-square or Fisher’s exact test was used to analyze categorical data. The two-proportion Z test was used to compare proportions. Logistic regression analysis was used to identify independent variables associated with outcome. Data are presented as mean (standard deviation) for normally distributed variables, median (interquartile range) for variables of not normal distribution, and number (percentage) for categorical variables. *p* < 0.05 was used to identify statistically significant differences.

## 3. Results

### 3.1. Population Characteristics

The questionnaires were properly completed by 223 patients (96.9%). Fourteen patients (6.3%) were unable to name the method of anesthesia they received. There were 209 questionnaires available for final analysis: 114 vs. 95 in the GA and SA groups, respectively. Baseline patient characteristics did not differ significantly between groups (Table 2).

Mean ages in the GA and SA groups were 53.41 and 50.15 years, respectively. Patient distribution by gender was similar. Most patients were ASA class II. More than 80% of the patients had previously experienced anesthesia, and 90% were highly satisfied. Preoperative pain scores were high in both groups, with self-assessed pain classified as severe by 86.1% and 81.5% patients in the GA and SA groups, respectively. There was one patient in each group who needed revision surgery (0.9% vs. 1.0%, respectively). The median hospitalization time was 4 days in both study groups.

### 3.2. Perioperative Pain Assessment

**Figure 1:**
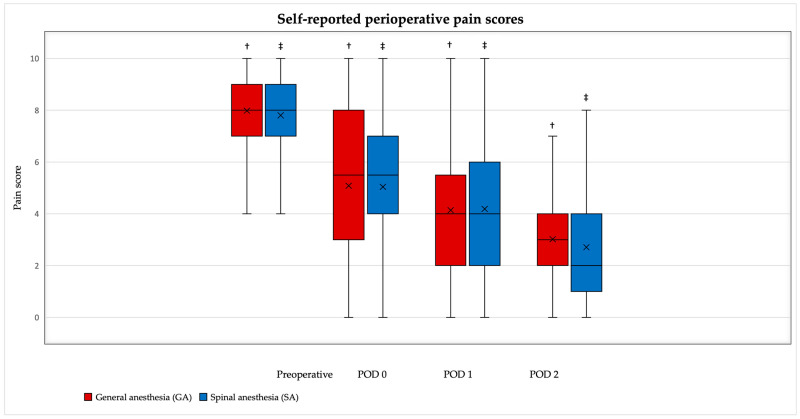
Self-reported perioperative pain scores. Data are presented as minimum, maximum, Q1, Q3, mean, and median. ^†^ *p* < 0.05 between sequential time points within GA group. ^‡^ *p* < 0.05 between sequential time points within SA group. Wilcoxon signed-rank tests.

Significant time-dependent postoperative pain decreases were found within groups (Figure 1); however, pain scores at distinct time points (preoperative, POD 0, POD 1 and POD 2) did not differ significantly between the GA and SA groups (Table 3).

There were no significant between-group differences regarding the distribution of patients by pain severity at different time points (Table 4).

### 3.3. Patient Satisfaction with Anesthesia

Mean overall satisfaction scores (OSSs) were high: 9.71 in the general anesthesia group and 9.75 in the spinal anesthesia group. Median OSS were equal: 10 (9.55–10) vs. 10 (9.7–10) and did not differ significantly between study groups (*p* = 0.95). Maximum satisfaction (10 out of 10 in all patient satisfaction questionnaire categories) was reported by 116 study patients, 65 (57%) vs. 51 (53.7%) patients in the GA and SA groups, respectively. Twenty-five patients (11.96%) reported OSS ≤ 9, 14 (12.28%) vs. 11 (11.58%) patients in the GA and SA groups.

When asked whether they would recommend the type of anesthesia they received, 99.1% of patients in the GA group and 95.8% of patients in the SA group responded positively (*p* = 0.12).

The ceiling effect of the patient satisfaction questionnaire had to be addressed. To identify areas for improvement, in-depth analysis of patients who did not express maximum satisfaction (N = 92) was performed. Forty-nine patients in the GA group (42.61%) and 43 patients in the SA group (45.26%) rated at least one question in the patient satisfaction questionnaire less than 10. Ratings assigned to different aspects of anesthesia care were compared between study groups (Table 5).

Patients in the SA group tended to assign better ratings to most aspects of anesthesia care, except for nausea and vomiting, but the differences were not statistically significant.

All patients in both study groups were highly satisfied (NRS ≥ 8) with the management of anxiety/fear; feelings of safety, relaxation, well-being; and kindness of medical staff (question Nos. 5, 6, 8, 9, 10).

**Figure 2:**
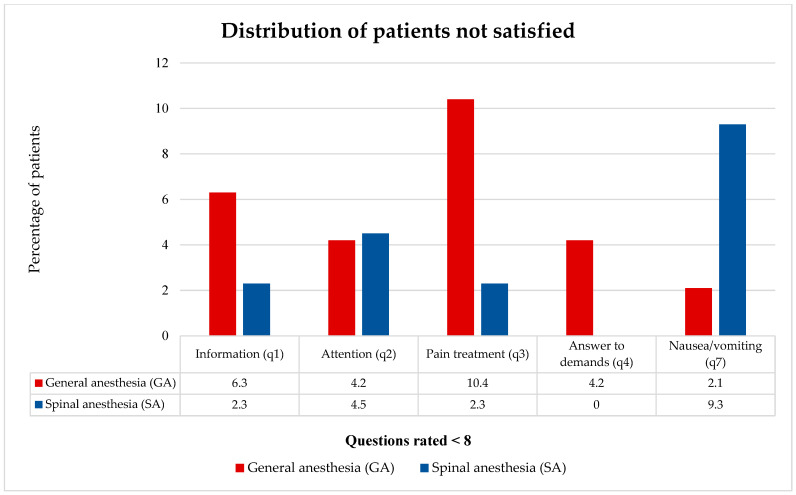
Distribution of patients not satisfied with certain aspects of anesthesia care. q, question (refer to Table 1).

The distribution of patients who were not fully satisfied (NRS < 8) with certain aspects of anesthesia care is presented in Figure 2.

There were more patients who were not fully satisfied with the provision of information, pain treatment, and responses to demands by the anesthesiologist in the GA group, whereas a higher number of SA group patients were not fully satisfied with the management of nausea and vomiting. However, statistical significance was not reached (Table 6).

### 3.4. Sources of Patient Discomfort

To identify other factors associated with patient satisfaction, patients were asked about sources of discomfort experienced in the operating room and postoperatively.

A total of 63 patients (30.1%) stated that they experienced discomfort in the operating room (31 (27.2%) vs. 32 (33.7%) patients in the GA and SA groups, respectively).

**Figure 3:**
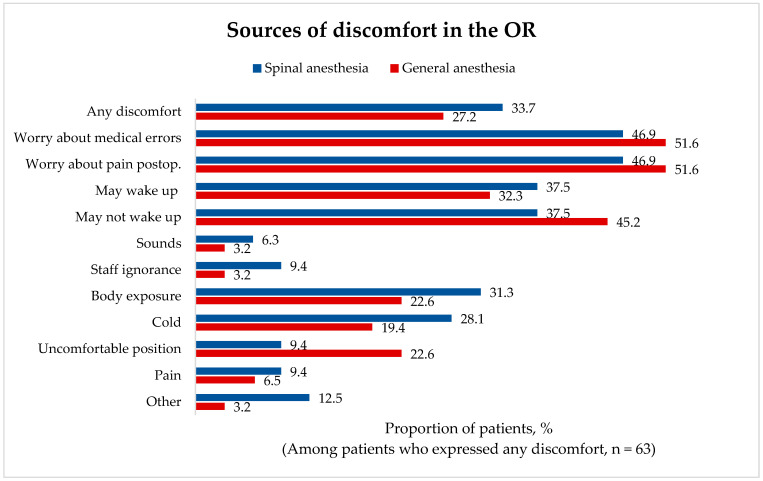
Sources of patient discomfort in the operating room.

Among those patients, anxiety associated with possible medical errors was the major concern, irrespective of the type of anesthesia. A larger proportion of patients were worried about waking up during the procedure in the SA group (37.5% vs. 32.3%), whereas in the GA group, more patients were worried about the possibility of not waking up (45.2% vs. 37.5%). External factors, including sounds, staff behavior, body exposure, and cold, were more prevalent sources of discomfort for SA group patients. Discomfort due to uncomfortable position in the OR was expressed by more patients in the GA group (6.1% vs. 3.2%) (Figure 3). The differences between study groups did not reach statistical significance.

**Figure 4:**
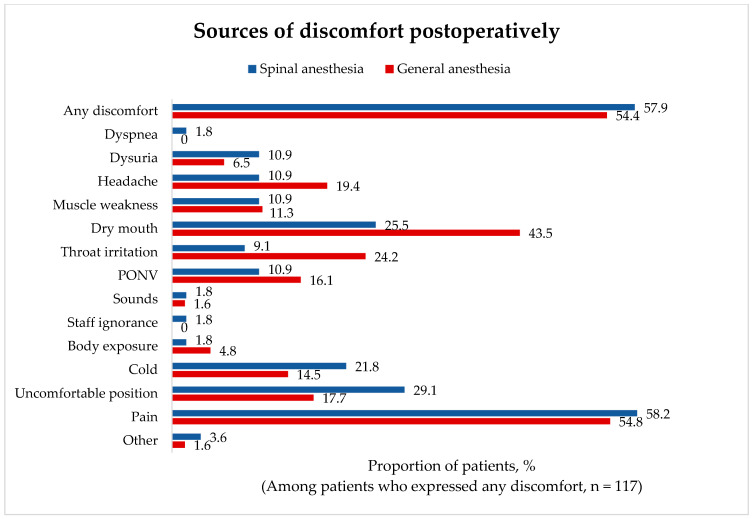
Sources of patient discomfort postoperatively. PONV, postoperative nausea and vomiting.

Postoperative discomfort was reported by 117 (56%) patients (62 (54.4%) vs. 55 (57.9%) patients in the GA and SA groups, respectively), and postoperative pain was a major concern in both groups. Throat irritation (*p* = 0.03) and mouth dryness (*p* = 0.04) were statistically significantly more common complaints in the GA group. There were no other statistically significant differences between study groups. GA group patients tended to have more headaches and postoperative nausea and vomiting, whereas SA group patients more commonly expressed discomfort due to dysuria, cold, and uncomfortable position (Figure 4).

### 3.5. Factors Affecting Patient Satisfaction with Anesthesia

To identify factors affecting patient satisfaction, univariate logistic regression analysis was performed. Failure to achieve maximum overall satisfaction (score < 10) was chosen as a reference categorical variable. Identified associations in the study patient population are presented in Table 7.

Univariate logistic regression analysis identified anxiety and nude body exposure as major preoperative factors associated with decreasing satisfaction with anesthesia. Postoperative factors associated with submaximal satisfaction were postoperative nausea and vomiting, headache, cold, mouth dryness, and pain. Interestingly, early postoperative pain (POD 0) did not influence overall patient satisfaction; an association was only found when pain persisted on POD 1 and POD 2. Adjustment for age did not influence primary results of the regression analysis.

The independent variables which were statistically significantly identified by univariate logistic regression analysis, were included in the subsequent age-adjusted multivariate logistic regression analysis. Anxiety about medical errors (OR 3.17; 95% CI 1.26–7.99, *p* = 0.015) and nude body exposure (OR 4.04; 95% CI: 1.04–15.80, *p* = 0.045) were identified as significant preoperative predictors of submaximal OSS. Postoperative nausea and vomiting (OR 8.02; 95% CI 1.58–40.79, *p* = 0.012), pain on POD 1 (OR 1.18; 95% CI 1.03–1.35, *p* = 0.02) and headache (OR 5.50; 95% CI: 1.42–21.41, *p* = 0.014) were identified as significant postoperative predictors of submaximal OSS.

There was no association between the type of anesthesia and overall satisfaction scores.

## 4. Discussion

In our prospective study, we investigated factors associated with patient satisfaction with anesthesia service during lumbar disc hernia surgery. Among the challenges faced by the scientific community are the lack of uniform definition of patient satisfaction and of a universally accepted evaluation tool [15]. We chose the questionnaire by Capuzzo et al. because it has been successfully implemented in previous studies and was suitable for both general and regional anesthesia [12,13]. Moreover, the use of a published assessment tool carries the advantage of presenting a reproducible study design. We additionally used a custom patient discomfort questionnaire to provide a more personalized approach to analyzing the sources of discomfort.

The first notable finding is that more than 80% of our patients undergoing lumbar disc hernia surgery rated their preoperative pain as severe. More than 60% of patients had symptoms lasting for more than 6 months, underlining the complexity of perioperative pain management in this patient population, in whom chronic pain syndrome may have developed.

Multiple randomized prospective studies have compared outcomes of lumbar spine surgery under regional versus general anesthesia [1,9,16,17], and authors have repeatedly reported lower postoperative pain scores in the spinal anesthesia group and linked them with patient satisfaction [9,16,17]. A meta-analysis of 3709 patients undergoing lumbar spine surgery under spinal versus general anesthesia showed that patients who received spinal anesthesia had significantly lower postoperative pain scores (mean difference: −2.80; 95% CI [−4.55 to −1.06], *p* = 0.002) [4].

In our study, the distribution of pain scores in the immediate postoperative period (POD 0) was slightly wider in the general anesthesia group; however, median pain scores and patient distribution by pain categories were comparable in the general anesthesia and spinal anesthesia groups at all time points. The absence of significant differences between GA and SA regarding immediate postoperative pain may be explained by the fact that the patients were asked to rate their overall pain score within the first 24 postoperative hours, rather than at multiple time points on the first postoperative day. However, our target outcome was the influence of perceived pain on overall satisfaction, and the analysis of not fully satisfied patients (NRS < 8) revealed more patients in the GA group, but the difference was not statistically significant. Thus, residual sensory block did not have a significant impact on postoperative pain perception on POD 0. Moreover, the pain score reported on POD 0 was not associated with patient satisfaction, implying that the anesthesia method was not a major contributor. It seems that the availability of contemporary general anesthetics and systemic analgesics must have diminished previously reported differences in favor of SA.

Postoperative pain management on postoperative days 1 and 2 was suboptimal in both groups and had adverse effects on patient satisfaction, as confirmed through logistic regression analysis. We cannot differentiate whether this finding is associated with failure to meet patient expectations from a surgical perspective, an anesthesiologic one, or both. Pain must be assessed continuously throughout the perioperative period (including subsequent postoperative days) and treated accordingly.

Demirel et al. [17] found better satisfaction scores in the regional anesthesia group (4.22 vs. 1.3 on a 5-point scale); Attari et al. [9] reported better satisfaction with spinal anesthesia on a dichotomous scale (Yes/No) with 100% satisfied with SA versus 67% satisfied with GA; and Vural et al. [16] reported 94% of patients satisfied with spinal as compared to 74% satisfied with general anesthesia.

In our study, patient overall satisfaction scores (OSSs), as well as satisfaction with different aspects of anesthesia care, were high, with medians of 9 to 10, and did not differ significantly between groups. Similar proportions of patients expressed maximum satisfaction and would have recommended the method of anesthesia they received (99.1% vs. 95.8%). Our results reflect that careful management of both anesthesia methods and adequate, patient-centered care may ensure equal patient satisfaction with perioperative care by the anesthesia team.

The fact that more than 50% of the patients gave maximum scores to every question in the satisfaction questionnaire resulted in a ceiling effect, reduced data variance and limited number of subjects in which areas for improvement could be investigated. This underlines the complexity of patient-reported satisfaction assessment which may be influenced by multiple factors including cultural differences and may make universal adaptation of available instruments even more complicated.

Anxiety and nude body exposure were major intraoperative factors associated with submaximal patient satisfaction. While the anxiety associated with possible medical errors may be hardly modifiable, the anxiety associated with possible failure to wake up, possible emergence during surgery, or possible pain can be managed with reassurance and careful information during the pre-anesthesia consult [18]. The analysis of dissatisfied patients (NRS < 8) revealed that more GA group patients expressed a lack of information about anesthesia and inadequate responses to their needs. Anesthesiologists may tend to inform patients before regional anesthesia more thoroughly, as compared to GA group patients, underlining the expectation of patients to be fully informed even if they will be unconscious intraoperatively. Discomfort due to nude body exposure increased the likelihood of submaximal OSS by more than 6 times; therefore, careful protection of patient privacy is crucial for patient satisfaction.

In addition to pain in POD 1 and POD 2 in the postoperative period, PONV, mouth dryness, headache, and cold were found to be associated with submaximal OSS. Logistic regression analysis confirmed that PONV increased the likelihood of submaximal OSS by 10 times. More patients in the GA group reported PONV, which is an expected finding as reported by other authors [1,17,19]. Interestingly, detailed analysis of not fully satisfied patients (NRS < 8) revealed more patients in the SA group. It is likely that PONV was less expected by patients in the SA group, and the presence of it had a more profound effect on the satisfaction score. PONV prophylaxis, therefore, should not be neglected in patients who undergo regional anesthesia.

Heidegger et al. discuss that a patient’s experience of adverse events, such as PONV and postoperative pain, is not the same as dissatisfaction with their management; moreover, empathic care by the medical staff may have more of an effect on patient satisfaction with care than a reduction in pain or PONV itself [15]. The effects of absolute pain scores and the rate of PONV on overall patient satisfaction with perioperative care may be overrated.

While oropharyngeal discomfort is an expected finding after GA, headaches were also more common following GA; thus, we can presume that lumbar puncture with an atraumatic G27 spinal needle did not have any significant influence on lumbar puncture-associated headache. The higher rate of headache in GA patients may be attributed to their static prone position in combination with anesthetic effects on cerebral hemodynamics, leading to transient intracranial pressure increase.

The fact that feeling cold was found to increase the likelihood of submaximal OSS by more than 2.5 times underlines the importance of maintaining normothermia during both regional and general anesthesia.

We must note that our study had several limitations. The study was single center, not randomized and had limited follow up period, therefore we cannot exclude the possibility of bias. Despite detailed analysis of satisfaction from the patient perspective, we did not evaluate surgeon satisfaction, perioperative turnover times, and costs, which may be key factors regarding the choice of anesthesia, given that both GA and SA were equally effective and well-tolerated by our patients. Another limitation is that our patient discomfort questionnaire was custom, and we could have missed some other significant factors important for patient satisfaction.

## 5. Conclusions

Both general anesthesia and spinal anesthesia were equally effective in terms of pain management, as well as from a patient satisfaction perspective. No major differences in sources of perioperative discomfort were found between groups. Patient information, anxiety management, and protection of patient privacy were major preoperative factors associated with patient satisfaction. In the postoperative period following lumbar disc hernia surgery, pain and PONV management must be equally addressed, irrespective of the anesthesia method used. Further efforts to develop optimal tools for patient satisfaction assessment are necessary.

## Figures and Tables

**Table 1:** The list of questions presented in the patient satisfaction questionnaire [12,13].

1.	How satisfied are you with the information given by the Anesthetist?
2.	How satisfied are you with the attention of the Anesthetist to you?
3.	How satisfied are you with treatment of pain at the site of surgery?
4.	How satisfied are you with answer to your demands in the operating room?
5.	How satisfied are you with the relief provided for feeling anxious or frightened?
6.	How satisfied are you with feeling safe perceived in the operating room?
7.	How satisfied are you with anesthesia, regarding vomiting and nausea?
8.	How satisfied are you with feeling relaxed when cared for by the Anesthetist?
9.	How satisfied are you with the feeling of wellbeing instilled in you by the Anesthetist?
10.	How satisfied are you with kindness of caregivers in the operating room?

**Table 2:** Baseline patient characteristics.

		All Patients (*n* = 223)	GA Group (*n* = 114)	SA Group (*n* = 95)	*p* Value
Gender, *n* (%)	Male	119 (53.4)	61 (53.5)	50 (52.6)	0.90
	Female	104 (46.6)	53 (46.5)	45 (47.4)	
Age, years; mean (SD)		52.05 (13.96)	53.41 (13.72)	50.15 (13.97)	0.09
General physical state (ASA), *n* (%)	I	52 (23.3)	22 (19.3)	26 (27.4)	0.43
	II	115 (51.6)	60 (52.6)	49 (51.6)	
	III	51 (22.9)	30 (26.3)	18 (18.9)	
	IV	5 (2.2)	2 (1.8)	2 (2.1)	
Prior anesthesia, *n* (%)		195 (87.4)	101 (88.6)	82 (86.3)	0.62
Satisfaction with prior anesthesia (NRS), median (Q1–Q3)		10 (9–10)	10 (9–10)	10 (9–10)	0.35
Maximum satisfaction with prior anesthesia (NRS = 10), *n* (%)		115 (64.2)	60 (61.2)	55 (67.9)	0.35
High satisfaction with prior anesthesia (NRS ≥ 8), *n* (%)		171 (89.5)	88 (89.8)	73 (90.1)	0.94
Hernia symptom duration, *n* (%)	<1 month	18 (8.1)	7 (6.1)	11 (11.6)	0.46
	1–6 months	62 (27.8)	32 (28.1)	26 (27.4)	
	6–12 months	45 (20.6)	21 (18.4)	20 (21.1)	
	>12 months	96 (43.3)	54 (47.4)	38 (42.9)	
Preoperative pain score, median (Q1–Q3)		8 (7–9)	8 (7–9)	8 (7–9)	0.69
Patient distribution on preoperative pain severity, *n* (%)	Mild (NRS 0–2)	6 (2.8)	2 (1.9)	4 (4.3)	0.52
	Moderate (NRS 3–6)	27 (12.6)	13 (12.0)	13 (14.1)	
	Severe (NRS 7–10)	181 (84.6)	93 (86.1)	75 (81.5)	

GA, general anesthesia; SA, spinal anesthesia; SD, standard deviation; NRS, numeric rating scale; Q, quartile. Student’s *t*-test, Chi-square test, or Mann–Whitney *U*-test as applicable.

**Table 3:** Median self-reported pain scores.

	GA Group (*n* = 114)	SA Group (*n* = 95)	*p* Value
Preoperative	8 (7–9) 101.98	8 (7–9) 98.77	0.69
POD 0	5.5 (3–8) 102.26	5.5 (4–7) 100.62	0.84
POD 1	4 (2–5.5) 99.75	4 (2–6) 101.40	0.84
POD 2	3 (2–4) 97.33	2 (1–4) 88.65	0.27

Data are expressed as median (Q1–Q3) and mean rank. GA, general anesthesia; SA, spinal anesthesia. Mann–Whitney *U*-test.

**Table 4:** Distribution of patients by pain categories at different time points.

	Preop. GA	Preop. SA	POD 0 GA	POD 0 SA	POD 1 GA	POD 1 SA	POD 2 GA	POD 2 SA
Mild	2 (1.9)	4 (4.3)	26 (24.1)	20 (21.3)	29 (26.6)	23 (25.3)	46 (44.2)	42 (51.2)
Moderate	13 (12)	13 (14.1)	45 (41.7)	46 (48.9)	64 (58.7)	54 (59.3)	52 (50)	35 (42.7)
Severe	93 (86.1)	75 (81.5)	37 (34.3)	28 (29.8)	16 (14.7)	14 (15.4)	6 (5.8)	5 (6.1)
*p* value *	0.516		0.584		0.974		0.605	

Data are expressed as number (%). Preop., preoperative; POD, postoperative day; GA, general anesthesia; SA, spinal anesthesia. * Chi-square test.

**Table 5:** Comparison of ratings between GA and SA groups after exclusion of patients with maximum satisfaction.

	GA, Median (Q1–Q3) Mean Rank	SA, Median (Q1–Q3) Mean Rank	*p* *
Information (q1)	9 (9–10) 41.95	10 (9–10) 51.47	0.06
Attention (q2)	9 (9–10) 43.05	10 (9–10) 50.26	0.15
Pain treatment (q3)	9 (8–9) 44.49	9 (8–10) 48.69	0.42
Answer to demands (q4)	10 (9–10) 42.65	10 (9–10) 50.70	0.09
Anxiety/fear management (q5)	10 (9–10) 44.28	10 (9–10) 47.92	0.45
Feeling of safety (q6)	10 (9–10) 44.21	10 (9–10) 49.00	0.31
Nausea/vomiting (q7)	10 (9–10) 47.44	10 (9–10) 44.40	0.54
Feeling of relaxation (q8)	10 (9–10) 44.54	10 (9–10) 48.64	0.36
Feeling of well-being (q9)	10 (9–10) 44.38	10 (9–10) 48.82	0.32
Staff kindness (q10)	10 (9.25–10) 45.94	10 (9.75–10) 47.11	0.78

GA, general anesthesia; SA, spinal anesthesia; q, question (refer to Table 1). * Mann–Whitney *U*-test.

**Table 6:** Distribution of patients not satisfied with certain aspects of anesthesia care.

	GA, *n* (%)	SA, *n* (%)	*p* *
Information (q1)	3 (6.3)	1 (2.3)	0.35
Attention (q2)	2 (4.2)	2 (4.5)	0.93
Pain treatment (q3)	5 (10.4)	1 (2.3)	0.11
Answer to demands (q4)	2 (4.2)	0 (0)	0.17
Nausea/vomiting (q7)	1 (2.1)	4 (9.3)	0.13

GA, general anesthesia; SA, spinal anesthesia, q, question (refer to Table 1). * Chi-square test.

**Table 7:** Univariate logistic regression analysis to identify factors affecting patient satisfaction with anesthesia.

In the Operating Room		Discomfort	Body Exposure	Worry May Not Wake Up	Worry May Wake Up	Worry About Pain	Worry About Medical Errors	
Overall patient satisfaction score < 10	OR	2.52	6.68	3.24	4.97	2.37	4.44	
	95% CI	1.37–4.61	1.86–24	1.34–7.84	1.76–14.06	1.09–5.16	1.88–10.47	
	*p*	0.003	0.004	0.009	0.003	0.03	0.01	
	aOR	2.50	7.19	3.44	5.06	2.63	4.58	
	95% CI	1.36–4.61	1.97–26.24	1.41–8.41	1.78–14.43	1.19–5.81	1.92–10.93	
	*p*	0.003	0.003	0.007	0.002	0.017		
**Postoperative Period**		**Discomfort**	**Cold**	**PONV**	**Dry Mouth**	**Headache**	**Pain Score (POD 1)**	**Pain Score (POD 2)**
Overall patient satisfaction score < 10	OR	2.04	2.76	10.10	2.03	7.82	1.20	1.17
	95% CI	1.17–3.58	1.07–7.15	2.23–45.69	1.01–4.05	2.21–27.78	1.05–1.36	1.02–1.35
	*p*	0.01	0.04	0.03	0.05	0.001	0.006	0.03
	aOR	2.02	2.60	12.51	2.27	9.02	1.21	1.20
	95% CI	1.15–3.56	1.0–6.82	2.67–58.49	1.12–4.63	2.49–32.68	1.06–1.38	1.04–1.39
	*p*	0.015	0.05	0.001	0.024	<0.001	0.004	0.013

Univariate logistic regression. All variables are represented as binary (Yes/No) except for pain scores. CI, confidence interval; aOR, age-adjusted odds ratio; PONV, postoperative nausea and vomiting; POD, postoperative day.

## Data Availability

The original contributions presented in this study are included in the article. Further inquiries can be directed to the corresponding author.

## Supplementary Materials

The following supporting information can be downloaded at: https://www.mdpi.com/article/10.3390/medicina62030524/s1, Supplementary material. Perioperative discomfort and pain questionnaire (original in native language).
