# Supplementary material for: Spinal Versus General Anesthesia for Lumbar Discectomy: Patient-Centered Analysis of Satisfaction with Anesthesia Service
Source: Medicina (Kaunas). 2026 Mar 12;62(3):524. doi: 10.3390/medicina62030524 (PMC13027776; doi:10.3390/medicina62030524)
Supplement: Supplementary file 1 [file medicina-62-00524-s001.zip › medicina-4165306-supplementary.pdf]

medicina MDPI

Supplementary material. Perioperative discomfort and pain questionnaire (original in native language).

<table>
<tr><td colspan="2">PREOPERATIVE</td></tr>
<tr><td>Your gender:
☐ Female
☐ Male</td><td>Your age:</td></tr>
<tr><td>Duration of your hernia symptoms in months:</td><td>Please rate your preoperative pain from 0 to 10 (0 = no pain; 10 = unbearable pain):</td></tr>
<tr><td>Have you previously experienced procedures with anesthesia?
☐ Yes
☐ No</td><td>Please rate your satisfaction with prior anesthesia from 0 to 10 (0 = complete dissatisfaction; 10 = maximum satisfaction):</td></tr>
<tr><td colspan="2">POSTOPERATIVE</td></tr>
<tr><td colspan="2">Please rate your postoperative pain from 0 to 10 (0 = no pain; 10 = unbearable pain):
☐ Day of surgery:
☐ Postoperative day 1:
☐ Postoperative day 2:</td></tr>
<tr><td>Please mark any discomfort experienced in the operating room:
☐ None
☐ Painful procedures
☐ Uncomfortable position of the body
☐ Cold
☐ Bare body exposure
☐ Indifference of the staff
☐ Unpleasant sounds in the operating room
☐ Worry that I may not wake up after the surgery
☐ Worry that I may wake up during the surgery
☐ Worry that I may feel pain
☐ Worry due to possible surgical errors
☐ Other:</td><td>Please mark any discomfort experienced after the surgery:
☐ None
☐ Pain
☐ Uncomfortable position of the body
☐ Cold
☐ Bare body exposure
☐ Indifference of the staff
☐ Unpleasant sounds in the ward
☐ Nausea / vomiting
☐ Throat soreness / discomfort
☐ Dry mouth
☐ Muscle pain / weakness
☐ Headache
☐ Urination problems
☐ Dyspnea
☐ Other:</td></tr>
<tr><td colspan="2">What type of anesthesia did you experience?
☐ General (I was fully unconscious throughout the surgery)
☐ Regional (A spinal puncture was performed before the start of surgery)
☐ Cannot answer</td></tr>
<tr><td colspan="2">Would you recommend the type of anesthesia you experienced to your close relative/friend?
☐ Yes
☐ No</td></tr>
</table>

https://doi.org/10.3390/medicina62030524
